# Cost-effectiveness analysis of first-line gefitinib plus anlotinib treatment for patients with stage IIIB–IV EGFR-mutated NSCLC in China

**DOI:** 10.3389/fpubh.2026.1840381

**Published:** 2026-07-10

**Authors:** Ping Chen, Yanhua Jiang, Yanli Chen, Shan Luo, Chuanmei Wu, Qiuyue Tang, Dandan Zhang, Yi Wang, Jiaqi Ma

**Affiliations:** Department of Thoracic Surgery, Sichuan Clinical Research Center for Cancer, Sichuan Cancer Hospital & Institute, Sichuan Cancer Center, University of Electronic Science and Technology of China, Chengdu, China

**Keywords:** anlotinib, cost-effectiveness, EGFR mutation, NSCLC, targeted therapy

## Abstract

**Background:**

The FL-ALTER study introduces a novel first-line therapeutic approach for patients with EGFR mutation-positive advanced non-small cell lung cancer (NSCLC). This study aimed to evaluate the cost-effectiveness of first-line gefitinib plus anlotinib for Chinese patients diagnosed with stage IIIB–IV EGFR-mutant NSCLC.

**Methods:**

A combined decision-tree and Markov model was constructed to predict 5-year cost-effectiveness using follow-up data from the FL-ALTER trial. Efficacy and safety inputs were derived from randomized clinical trials, whereas cost and utility values were sourced from published literature. Incremental cost-effectiveness ratios (ICERs) were calculated from the perspective of the Chinese healthcare system. Furthermore, scenario analyses were performed to evaluate the economic impact of patient assistance programs (PAPs) and drug price negotiations.

**Results:**

Compared with gefitinib plus placebo, first-line gefitinib plus anlotinib yielded an additional 0.16 quality-adjusted life years (QALYs), with an incremental cost-effectiveness ratio (ICER) of $196,652.56/QALY. Deterministic sensitivity analysis revealed that the utility of progression-free survival and the cost of anlotinib were the most influential parameters driving the model outcomes. Probabilistic sensitivity analysis indicated that the combination regimen had no cost-effectiveness advantage at the current Chinese willingness-to-pay (WTP) threshold of $38,043.34/QALY.

**Conclusion:**

Within the Chinese healthcare system, first-line gefitinib plus anlotinib is not cost-effective for advanced EGFR-mutated NSCLC at a WTP threshold of $38,043.34/QALY. However, implementing a PAP or price negotiation for anlotinib could effectively lower incremental costs, rendering this regimen a more economically viable clinical option.

## Introduction

Lung cancer remains the leading cause of cancer-related mortality in China, accounting for 22.0% of newly diagnosed cancer cases and 28.50% of all cancer-related deaths in 2022 (1). The mortality rate among males is 1.69 times higher than that among females, highlighting a disproportionately poorer prognosis in men (1). Economically, the burden of lung cancer was estimated to reach 40.40 billion US dollars by 2025 and is projected to further escalate to 53.4 billion US dollars by 2030. These figures represent 0.13% and 0.14% of the national gross domestic product (GDP) for the respective years, underscoring the pressing need for policymakers to integrate effective budgetary measures within healthcare systems to alleviate this financial strain (2). Epidermal growth factor receptor (EGFR) mutations occur in 10%−26% of patients with non-small cell lung cancer (NSCLC) and are particularly prevalent in Asian populations (3, 4), rendering these alterations crucial molecular targets for targeted lung cancer therapy. Epidermal growth factor receptor tyrosine kinase inhibitors (EGFR-TKIs) represent the standard-of-care first-line treatment for EGFR mutation-positive NSCLC, significantly reducing the risk of disease progression and metastasis (5, 6); however, the inevitable emergence of acquired resistance remains a formidable challenge in oncological research (7), severely limiting the long-term efficacy of these targeted therapies (8).

In recent years, targeted therapies have significantly improved clinical outcomes for patients with advanced EGFR mutation-positive NSCLC. However, the inevitable development of resistance has prompted researchers to actively investigate combination strategies aimed at enhancing therapeutic efficacy and prolonging patient survival (9). Despite their clinical promise, these combination therapies may escalate toxicities and treatment complexity. Although such combination regimens have not yet been established as the standard of care, they offer a viable therapeutic alternative for selected patients. Consequently, more convenient, efficacious, and less toxic combination regimens remain urgently required to address the unmet medical needs of patients with EGFR-mutated NSCLC.

Anlotinib, a domestically developed multitargeted tyrosine kinase inhibitor, has been approved in China for treating advanced NSCLC and soft tissue sarcoma since its launch in 2018. In the multicenter, phase III FL-ALTER trial (NCT04028778), first-line gefitinib plus anlotinib significantly prolonged the median progression-free survival (PFS) to 14.8 months in patients with stage IIIB–IV EGFR-mutated NSCLC, compared with 11.2 months for the gefitinib plus placebo group. This clinical benefit represented a significant risk reduction in disease progression [hazard ratio (HR) = 0.64; 95% confidence interval (CI): 0.48–0.86; *p* = 0.003]. Similarly, the median duration of response was prolonged with the combination therapy compared to the control arm (12.48 months vs. 9.46 months). Regarding safety, although the incidence of grade ≥3 treatment-related adverse events was higher in the combination group than in the control group (49.70% vs. 31.00%), the predominantly reported event was hypertension, and the overall safety profile remained clinically manageable and tolerable. From a biomarker perspective, this study also explored the potential beneficiary population, aligning with the concepts of precision medicine and providing critical guidance for personalized clinical decisions. Furthermore, this all-oral combination regimen not only facilitates patient adherence and compliance but also expands the therapeutic repertoire for patients with advanced NSCLC, effectively delaying the onset of drug resistance.

This study evaluated the cost-effectiveness of first-line gefitinib plus anlotinib for Chinese patients with stage IIIB–IV EGFR-mutated NSCLC within the context of the national healthcare system. Despite the substantial clinical efficacy demonstrated by this combination therapy, its high treatment-associated costs impose a significant financial toxicity on patients. This economic strain potentially compromises patient adherence and long-term quality of life (2). Furthermore, these clinical challenges are further exacerbated by real-world constraints, including the heterogeneous distribution of healthcare resources and regional economic disparities (10, 11). Consequently, this study seeks to investigate approaches for enhancing the accessibility and long-term economic sustainability of this advanced treatment option. To our knowledge, this is the first cost-effectiveness analysis to evaluate the first-line gefitinib plus anlotinib regimen. By addressing this critical gap in pharmacoeconomic evidence, our findings provide a scientific and quantitative benchmark to mitigate financial toxicity for patients, thereby guiding personalized clinical decision-making and informing dynamic pricing strategies within the national health insurance system.

## Methods

As a pharmacoeconomic evaluation based entirely on published literature data and mathematical modeling, this study did not involve human participants, the use of human tissues, or subject recruitment. Consequently, institutional review board (or ethics committee) approval and patient informed consent were not required. All economic analyses were conducted in strict accordance with the China Guidelines for Pharmacoeconomic Evaluations and relevant academic research norms.

### Model structure

**Figure 1:**
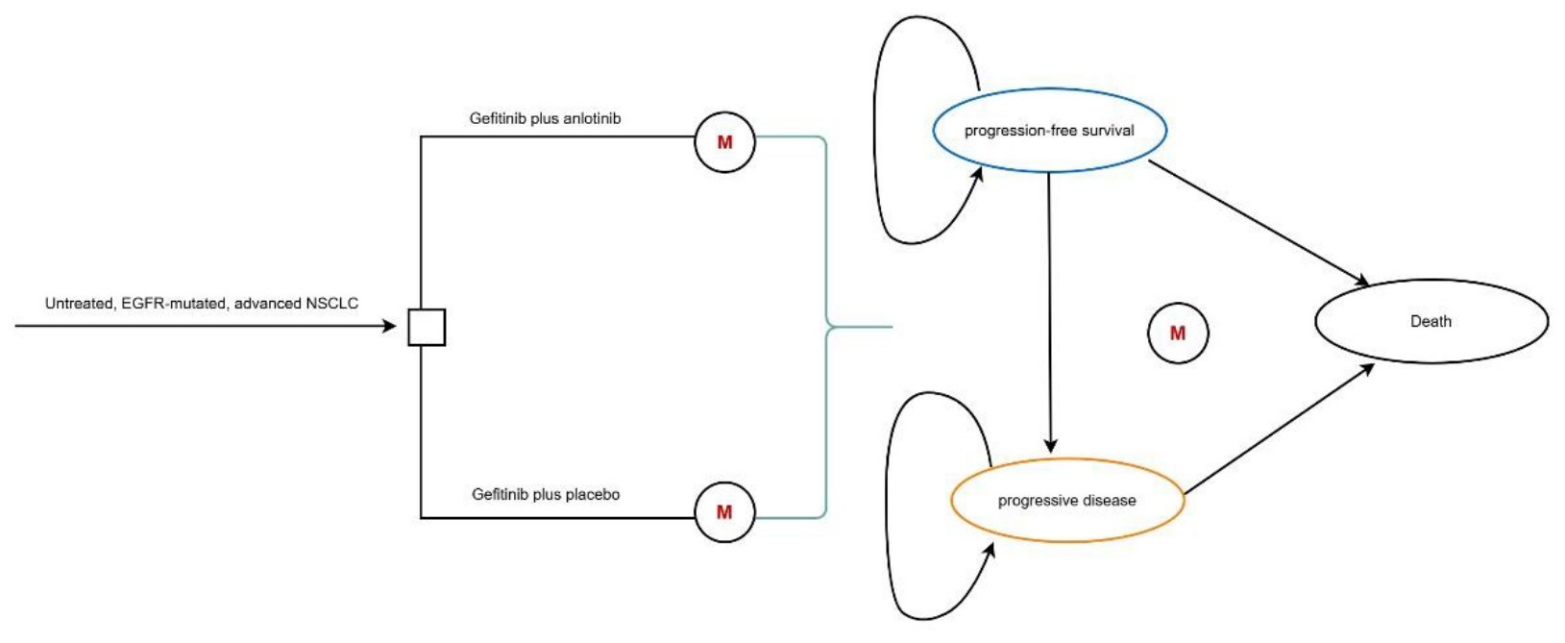
Markov state-transition model for untreated, EGFR-mutated, advanced non-small cell lung cancer in China. M, Markov node; NSCLC, non-small cell lung cancer.

To evaluate the clinical and economic outcomes of first-line gefitinib plus anlotinib for Chinese patients with stage IIIB–IV EGFR-mutated NSCLC, a decision tree and Markov model were implemented using TreeAge Pro 2022 software from the perspective of the Chinese healthcare system. Baseline characteristics of the target population in this model were based on the FL-ALTER trial. The primary inclusion criteria were histologically or cytologically confirmed locally advanced or metastatic NSCLC, documented sensitizing EGFR mutations, no prior systemic treatment for advanced NSCLC, and an Eastern Cooperative Oncology Group (ECOG) performance status of 0 or 1. Patients were excluded based on the following criteria: mixed small cell lung cancer histology, symptomatic brain metastases, tumor lesions within ≤ 5 mm of a major blood vessel, or central tumors invading local major blood vessels. Based on the FL-ALTER trial data, the model comprised three mutually exclusive health states: progression-free survival (PFS), progressive disease (PD), and death. The model was simulated with a cycle length of 21 days over a 5-year base-case time horizon (Figure 1). In the base-case analysis, the model projected outcomes over a 5-year period. However, considering the limited follow-up duration of the clinical trial and the potential for long-term patient survival, the time horizon was extended to 10 years in a scenario analysis to evaluate its impact on the robustness of the results. State transitions were driven by probabilities derived from the FL-ALTER trial, assuming that patients in the PD state would receive subsequent chemotherapy, targeted therapy, or immunotherapy (9, 12). Direct medical costs were evaluated to compare two distinct treatment arms: first-line gefitinib combined with anlotinib vs. gefitinib combined with placebo. The model estimated life-years (LYs), quality-adjusted life-years (QALYs) (13), and cumulative costs. Both costs and utilities were discounted at a 4.5% annual rate, as recommended by the China Guidelines for Pharmacoeconomic Evaluations (2025 Edition), applying an exchange rate of 1 USD = 7.07 RMB. The incremental cost-effectiveness ratio (ICER) was calculated to measure the incremental cost per additional QALY. In accordance with established guidelines, the willingness-to-pay (WTP) threshold was set at three times the per capita gross domestic product (GDP) of China ($38,043.34/QALY) (14). All model parameters were populated using aggregate data obtained exclusively from publicly available, published literature (15).

### Treatment details

The FL-ALTER study enrolled patients aged 18–75 years with untreated stage IIIB or IV NSCLC harboring EGFR exon 19 deletions (19del) or exon 21 L858R mutations. Eligible participants were required to have an ECOG performance status of 0 or 1, measurable disease per the Response Evaluation Criteria in Solid Tumors (RECIST) version 1.1, and adequate organ function. Patients were randomly allocated to either the investigational arm (receiving gefitinib plus anlotinib) or the control arm (receiving gefitinib plus placebo). Patients in the investigational arm received oral gefitinib (250 mg, once daily continuously) combined with oral anlotinib (12 mg, once daily on Days 1–14 of each 21-day cycle) until disease progression. Conversely, patients in the control arm received the identical regimen of gefitinib monotherapy supplemented with a matching placebo. Baseline safety data indicated that the incidence of grade ≥3 treatment-related adverse events (TRAEs) was 49.7% in the combination arm, compared with 31.0% in the control arm. Within the combination arm, hypertension represented the primary grade ≥3 adverse event, with no unexpected safety signals observed, and the overall safety profile was deemed clinically tolerable (9). In the Markov model, all simulated patients in the PFS state received therapies in strict adherence to the specific regimens and dosages specified in the FL-ALTER trial protocol.

In China, ongoing healthcare reforms focus on enhancing drug accessibility and alleviating patient financial burdens through patient assistance programs (PAPs) and national drug price negotiations (16), aligning with global paradigms of rational healthcare resource allocation. Within this clinical and economic landscape, this study evaluates the cost-effectiveness of first-line gefitinib plus anlotinib for patients with advanced EGFR-mutated NSCLC, incorporating and building upon scenarios previously identified in the literature (17–19).

To reflect real-world affordability, the model incorporated a patient assistance program (PAP) initiated in 2018, which aimed to alleviate the economic burden and enhance the quality of life for patients with advanced stage IIIB–IV EGFR-mutated NSCLC. The PAP framework comprised an initial assistance phase (10 paid cycles followed by 8 complimentary cycles) and a subsequent assistance phase (5 paid cycles followed by 13 complimentary cycles). Under the PAP framework, patients initially received up to 8 complimentary cycles of anlotinib following 10 self-funded cycles, subject to clinical evaluation and eligibility verification. In the subsequent phases, this dynamic adjusted to a repeating cycle of 5 self-funded cycles followed by up to 13 complimentary cycles (20). Accordingly, this study evaluated the cost-effectiveness of first-line gefitinib plus anlotinib for Chinese patients with advanced stage IIIB–IV EGFR-mutated NSCLC specifically under the scenario of PAP implementation.

### Transfer probability data

In this study, a Markov model was employed to simulate patient trajectories starting from the PFS state, with patients either remaining in this state or transitioning to PD or death based on data from the FL-ALTER trial (21, 22). Regarding safety, only severe or serious adverse events (SAEs) with an incidence of ≥5% were incorporated into the cost estimation (23). To project long-term survival, progression-free and overall survival curves were extrapolated using the algorithms described by Guyot et al. Pseudo-individual patient data (IPD) were reconstructed and subsequently fitted via a parametric accelerated failure time (AFT) model to circumvent the constraints of the proportional hazards (PH) assumption (23). Data points from the PFS and OS curves were extracted utilizing GetData Graph Digitizer (version 2.26). The generated data were processed within the R environment, and parametric survival distributions were fitted using Stata (version 16.0). Model goodness-of-fit was rigorously assessed based on the Akaike Information Criterion (AIC) (Table 1). The specific survival models and estimated parameters for both the gefitinib plus anlotinib and gefitinib plus placebo cohorts are detailed in Table 2.

**Table 1:** Summary of the goodness of statistical fit for the Kaplan-Meier curve in the NCT04028778 trial.

	Exponential	Weibull	Gompertz	Lognormal	Loglogistic
Gefitinib plus anlotinib PFS curve for patients with treatment-naïve, EGFR-mutated, advanced NSCLC					
AIC	299.4093	269.9641	285.7365	266.3086	262.9701
Gefitinib plus anlotinib OS curve for patients with treatment-naïve, EGFR-mutated, advanced NSCLC					
AIC	195.0669	182.5316	183.5161	187.7555	183.2513
Gefitinib plus placebo PFS curve for patients with treatment-naïve, EGFR-mutated, advanced NSCLC					
AIC	296.5234	250.8953	274.6813	240.9064	237.9161
Gefitinib plus placebo OS curves for patients with treatment-naïve, EGFR-mutated, advanced NSCLC					
AIC	191.8816	188.7469	189.2314	189.8958	189.1870

**Table 2:** Model parameters: baseline values, ranges, and distributions for sensitivity analysis.

Parameter	Value	Range	Distribution	Ref
Survival				
Gefitinib plus anlotinib group				
Loglogistic curve of PFS for untreated patients with EGFR-mutated advanced non-small cell lung cancer	λ = 0.064221; γ = 0.421351	-	-	(9)
Weibull curve of OS for untreated patients with EGFR-mutated advanced non-small cell lung cancer	λ = 0.001094; *p* = 1.836178	-	-	(9)
Gefitinib plus placebo group				
Loglogistic curve of PFS for untreated patients with EGFR-mutated advanced non-small cell lung cancer	λ = 0.084306; γ = 0.361425	-	-	(9)
Weibull curve of OS for untreated patients with EGFR-mutated advanced non-small cell lung cancer	λ = 0.003125; *p* = 1.460047		-	(9)
Costs ($)				
Gefitinib price (250 mg)	22.56	16.92–28.20	Lognormal	(27)
Anlotinib price per mg	6.90	5.18–8.63	Lognormal	(28)
Follow-up per cycle	146.51	109.88–183.14	Lognormal	(28)
Treatment after progression per cycle	1,230.60	922.95–1,538.26	Lognormal	(28)
Terminal care	2,520.43	1,890.32–65,531.23	Lognormal	(27)
Hypertension	16.96	12.72–21.20	Lognormal	(28)
ALT/AST increased	67.40	50.55–84.25	Lognormal	(26)
Utilities				
PFS	0.60	(0.48, 0.72)	Beta	(28)
PD	0.56	(0.45, 0.67)	Beta	(28)
Hypertension	−0.12	(−0.09, −0.15)	Beta	(28)
**Risk for treatment-related AEs**				
Gefitinib plus anlotinib group				
Hypertension	29.68%	22.26%−37.10%	Beta	(9)
ALT increased	6.45%	4.83%−8.06%	Beta	(9)
Gefitinib plus placebo group				
Hypertension	5.20%	3.90%−6.50%	Beta	(9)
ALT increased	12.30%	9.22%−15.37%	Beta	(9)
AST increased	7.10%	5.32%−8.87%	Beta	(9)
Other				
Discount rate (%)	4.5	0–8	Fixed in PSA	Guidelines

PFS, progression-free survival; PD, progressive disease.

In this study, survival parameters and mathematical functions for each PFS and OS curve were estimated based on standard formulation frameworks compatible with TreeAge Pro (https://www.treeage.com/) and Stata. Consequently, time-dependent transition probabilities for the Markov processes were derived. The model implementation was structured based on the following core assumptions:

(1) The transition probability from the PFS state to death is assumed to be equivalent to the background natural mortality rate of the Chinese population aged ≥59 years, as reported by the World Health Organization (WHO).
(2) The transition probability from PFS to PFS is given by S(t)/S(t-μ), where μ represents the cycle length of the Markov process.
(3) PFS to PD probability of 1–*P*_*PFS to Death*_ – *P*_*PFS to PFS*_

We also calculated the probability of transition from survival to survival (P _StoS_) and derived the probability of transition from PD to PD using the following formula:


$$\begin{array}{l} \left[(n_{PFS+}n_{PD})*P_{S\;to\;S}-n_{PFS}*P_{PFS\;to\;PFS}-n_{PFS}*P_{PFS\;to\;PD}\right]/n_{PD} \end{array}$$

The probability of transition from PD to death is as follows:


$$\begin{array}{l} \left(P_{PD\;to\;Death}\right)=1-P_{PD\;to\;PD} \end{array}$$

where nPFS and nPD represent the number of patients with PFS and PD, respectively, in the previous cycle (24).

### Costs and utility values

From the perspective of the Chinese healthcare system, direct medical costs encompassed drug procurement, routine follow-up care, management of SAEs with an incidence of ≥5%, subsequent treatments following disease progression, and end-of-life care (25–27). For hypertension, the most common adverse event, cost estimation was calculated by multiplying its incidence by the corresponding management cost. The relevant financial data were sourced from authoritative public hospitals or published literature. All monetary values were converted into US dollars at an exchange rate of 7.07 RMB per USD, and subsequent treatment expenditures were referenced from established economic evidence. Costs associated with treatment-related SAEs exceeding a 5% incidence threshold were included and assumed to be incurred entirely within the first model cycle (9). Health state utility values for stage IIIB–IV EGFR-mutated NSCLC were sourced from published literature (25) and seamlessly integrated into the model framework. Furthermore, the parameter uncertainties regarding both medical expenditures and utility scores were systematically evaluated through comprehensive sensitivity analyses.

### Sensitivity analyses

To evaluate the impact of parameter uncertainty on the model outcomes, a deterministic sensitivity analysis (DSA) was conducted utilizing TreeAge Pro 2022. Model variables were systematically varied within their 95% confidence intervals or across a range of ±25% from the baseline values where clinical data were unavailable. Additionally, to assess the joint stability of the findings, a probabilistic sensitivity analysis was performed via 100,000 monte carlo simulations for two distinct scenarios.

## Results

### Base-case analysis: combination therapy increases QALYs and total costs

From the perspective of the Chinese healthcare system, the 5-year model projection revealed that, compared with the gefitinib plus placebo cohort, first-line treatment with gefitinib plus anlotinib for stage IIIB–IV EGFR-mutated NSCLC conferred an incremental gain of 0.24 LYs and 0.16 QALYs. Notably, these clinical benefits remained robust irrespective of whether a PAP or a national drug price negotiation scenario was implemented. Meanwhile, the 10-year model projection demonstrated that the combination therapy conferred an incremental gain of 0.38 LYs and 0.23 QALYs compared with the control arm (Table 3). The base-case results summarized in Table 3 indicate that in the baseline scenario (in the absence of a PAP or national drug price negotiation), the 5-year and 10-year ICERs were $196,652.56/QALY and $151,745.68/QALY, respectively, both of which were well above the designated willingness-to-pay (WTP) threshold. Conversely, in Scenario 1 (with PAP implementation), the 5-year and 10-year ICERs for the gefitinib-anlotinib cohort dropped precipitously to $6,863.62/QALY and $3,932.37/QALY, respectively, falling well below the Chinese willingness-to-pay (WTP) threshold of $38,043.34/QALY. These findings suggest that while the combination regimen is not cost-effective under baseline unassisted conditions, it becomes a highly cost-effective clinical option when supported by a PAP. Furthermore, because the long-term survival projections for the price negotiation scenarios (Scenarios 2–1 to 2–3) mirrored those of the base-case analysis, and their economic viability had been thoroughly validated, the 10-year ICER data for these negotiation scenarios were intentionally omitted to avoid statistical redundancy.

**Table 3:** Base-case analyses of costs for gefitinib plus anlotinib vs. gefitinib plus a placebo with incremental changes in China.

Strategies	Total cost $		LYs		QALYs		ICER ($/QALY)	
	5 years	10 years	5 years	10 years	5 years	10 years	5 years	10 years
No PAP and price negotiation								
Gefitinib plus anlotinib	73,989.15	94,391.58	2.74	3.58	1.60	2.07	196,652.56	151,745.68
Gefitinib plus placebo	42,524.74	59,186.58	2.50	3.20	1.44	1.84	–	

LYs, life years; QALYs, quality-adjusted life years.

### Sensitivity analyses: ICER remains robust against parameter variations

**Figure 2:**
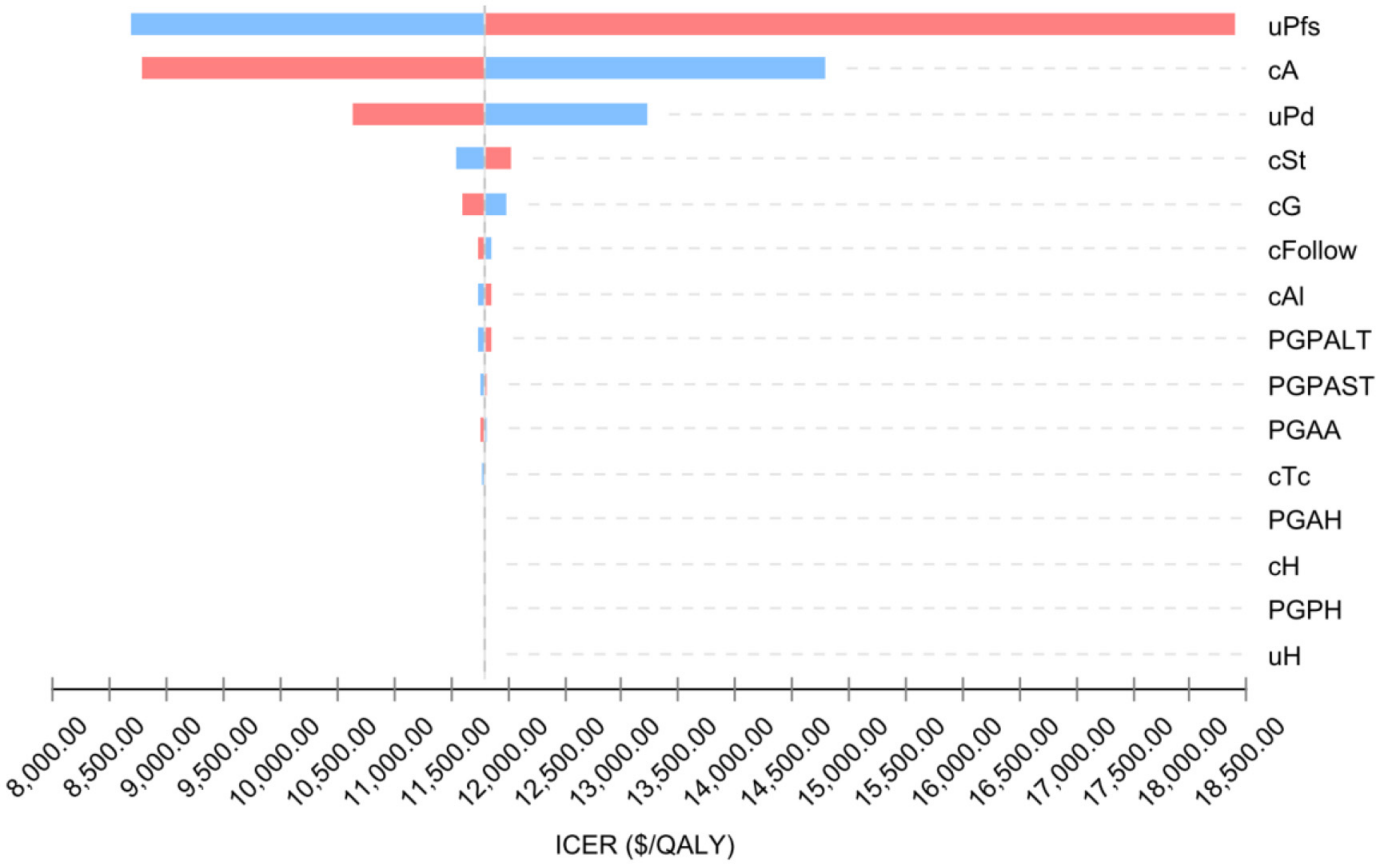
Deterministic sensitivity analysis of gefitinib plus anlotinib vs. gefitinib plus placebo. Key parameters significantly influencing the model outcomes included the utility of progression-free survival (uPfs), cost per milligram of anlotinib (cA), utility of disease progression (uPd), cost of subsequent treatment after disease progression (cSt), cost per 25 mg of gefitinib (cG), probability of elevated alanine aminotransferase (ALT) levels in the gefitinib plus a placebo group (PGPALT), probability of elevated aspartate aminotransferase (AST) levels in the gefitinib plus a placebo group (PGPAST), probability of elevated ALT levels in the gefitinib plus anlotinib group (PGAA), cost of palliative care in end-stage disease (cTc), probability of hypertension in the gefitinib plus anlotinib group (PGAH), cost of treating hypertension (cH), and probability of treating hypertension in the gefitinib plus a placebo group (PGPH).

**Figure 3:**
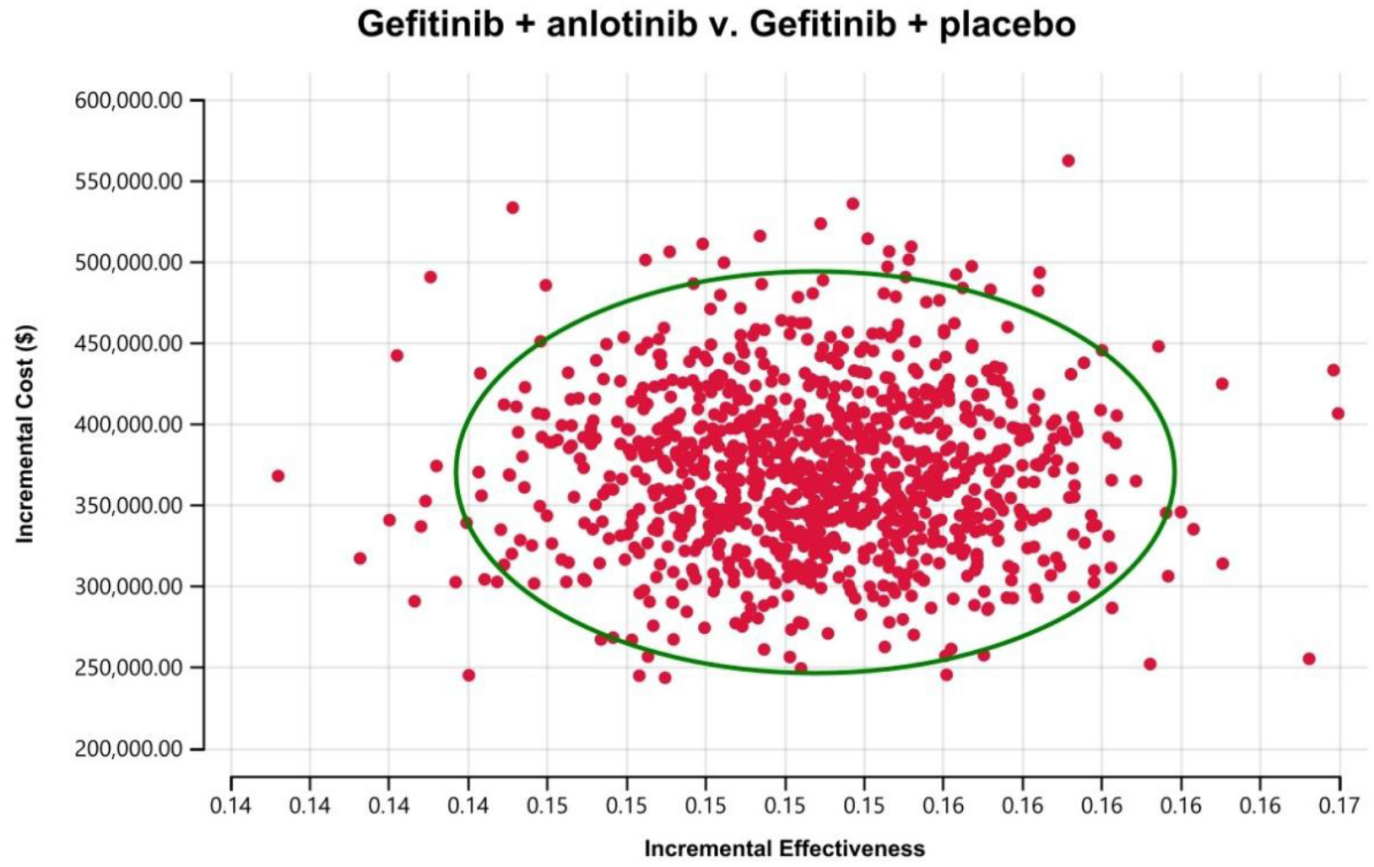
Cost-effectiveness plane for the gefitinib-anlotinib vs. gefitinib-placebo regimens (WTP = $38,043.34/QALY). The *X*- and *Y*-axes represent incremental QALYs and incremental costs, respectively. The ellipse indicates the 95% confidence interval. At the Chinese WTP threshold, all simulation iterations (red dots) are positioned above the threshold line, demonstrating that the combination lacks a cost-effectiveness advantage.

**Figure 4:**
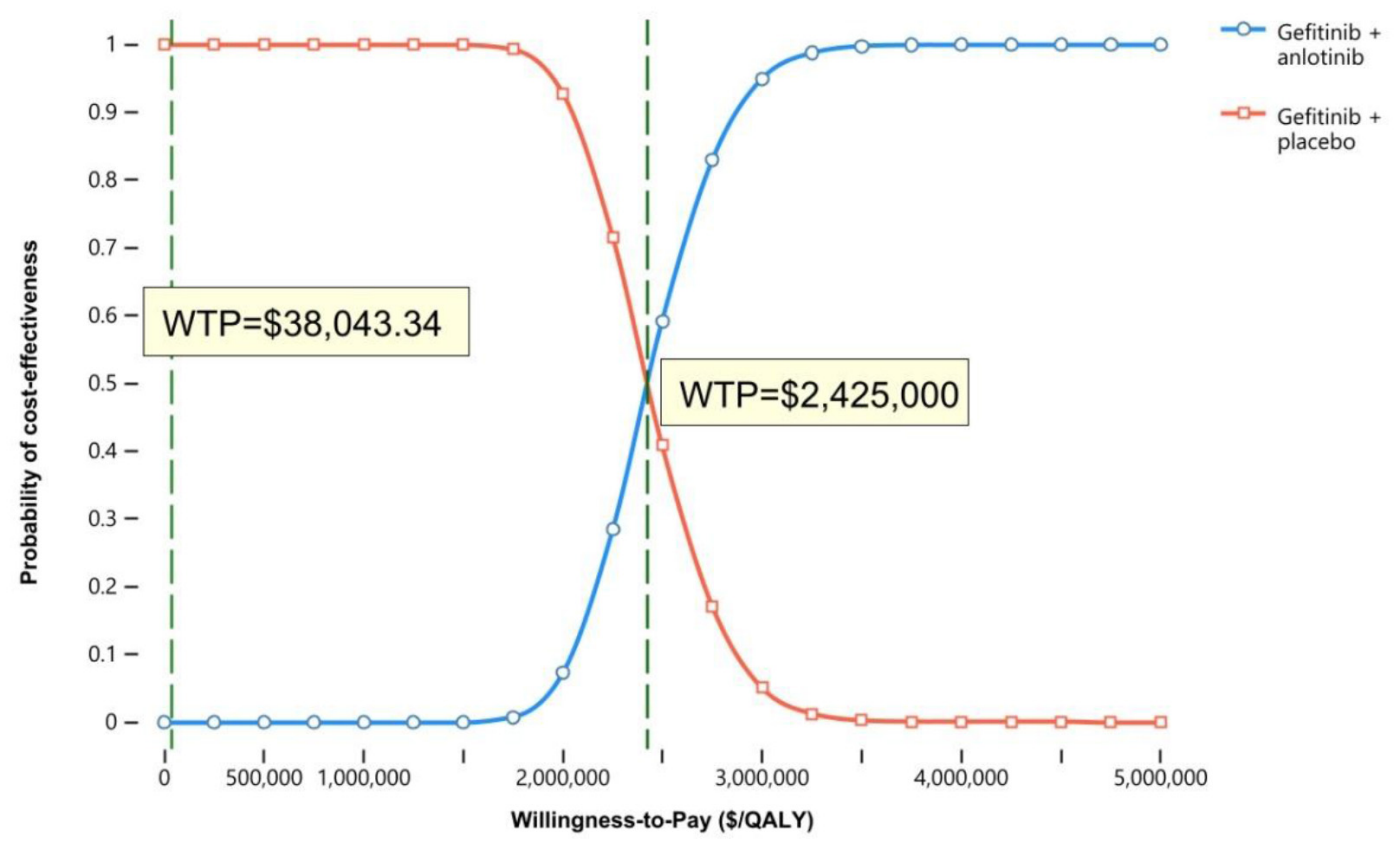
Cost-effectiveness acceptability curves in the base-case analysis: gefitinib plus anlotinib vs. gefitinib plus placebo.

In the deterministic one-way sensitivity analysis, each model parameter was varied individually within its predefined plausible range (± 25% of the baseline value or the 95% confidence interval) to recalculate the corresponding ICERs. The Tornado diagram illustrated that the model outcomes were most sensitive to variations in the utility of PFS and the cost of anlotinib, both of which exerted the most pronounced impacts on the ICER across the entire model (Figure 2). The remaining parameters exerted minimal influence on the model outcomes, and the ICERs were relatively insensitive to the management expenditures of adverse events. To evaluate the collective impact of parameter uncertainty, a probabilistic sensitivity analysis (PSA) was performed. As illustrated in the cost-effectiveness scatter plot (Figure 3), the distribution of simulated outcomes was mapped to assess robustness. The analysis revealed that at the designated Chinese WTP threshold of $38,043.34/QALY, the gefitinib-anlotinib combination therapy had a 0% probability of being cost-effective compared with gefitinib plus placebo (Figure 4). Notably, at a WTP threshold of approximately $2,425,000 per QALY, both regimens exhibited a cost-effectiveness acceptability probability of nearly 50%, indicating that the two therapies achieve mathematical equivalence at this high threshold inflection point (Figure 4). However, the acceptability probability of the gefitinib-anlotinib combination therapy climbs to 100% at realistic thresholds if a PAP is integrated or if the baseline price of anlotinib is reduced to $\le$20% of its original cost.

## Discussion

ersus gefitinib plus placebo for the treatment of stage IIIB/IV NSCLC from the perspective of the Chinese healthcare system. The findings indicate that, compared with the monotherapy control, the addition of anlotinib to gefitinib does not achieve cost-effectiveness as a first-line targeted therapeutic regimen within the evaluated patient population under baseline unassisted conditions. In Scenario 1 and Scenarios 2–1 to 2–3, an in-depth policy-driven analysis was performed, incorporating PAPs and drug price negotiations tailored to the Chinese healthcare ecosystem. The primary objective of this study was to evaluate the long-term cost-effectiveness—rather than merely clinical efficacy—of the first-line gefitinib-anlotinib combination for Chinese patients diagnosed with stage IIIB–IV EGFR-mutant NSCLC. Consequently, this economic evaluation provides a robust scientific foundation to inform value-based clinical decision-making and price-setting strategies (9). Our findings indicate that, compared with gefitinib plus placebo, the gefitinib-anlotinib combination therapy is cost-effective with an ICER below the preset WTP threshold under the anlotinib PAP, directly reducing the financial burden on patients during treatment. To align with the timeline of the FL-ALTER trial, this model adopted the 2020 National Reimbursement Drug List (NRDL) prices as the baseline. The 29.23% represents the comprehensive net out-of-pocket proportion actually borne by patients for anlotinib following national price negotiations and medical insurance reimbursement. This implies that even with the healthcare insurance system covering approximately 70.77% of the costs (Table 4), the ICER of this first-line combination regimen remains above the WTP threshold, indicating it is not cost-effective under current conditions. Furthermore, to adequately account for recent price updates and the potential trend of further price reductions for anlotinib, this study prospectively evaluated its long-term economic impact through Scenario 2–3 (simulating a price reduction to 20% of its original price).

**Table 4:** Scenario analysis of the cost-effectiveness of gefitinib plus anlotinib vs. gefitinib plus placebo under varying anlotinib price reduction rates in China.

Strategies and scenarios	Total cost $		LYs		QALYs		ICER ($/QALY)	
	5 years	10 years	5 years	10 years	5 years	10 years	5 years	10 years
Scenario 1: with PAP								
Gefitinib plus anlotinib	43,622.92	60,098.89	2.74	3.58	1.60	2.07	6,863.62	3,932.37
Gefitinib plus placebo	42,524.74	59,186.58	2.50	3.20	1.44	1.84	–	–
Scenario 2–1: price reduction to 30%								
Gefitinib plus anlotinib	51,549.06	-	2.74	-	1.60	-	56,403.68	-
Gefitinib plus placebo	42,524.74	-	2.50	-	1.44	-	-	
Scenario 2–2: price reduction to 29.23%								
Gefitinib plus anlotinib	51,302.21	-	2.74	-	1.60	-	54,859.18	-
Gefitinib plus placebo	42,524.74	-	2.50	-	1.44	-	-	
Scenario 2–3: price reduction to 20%								
Gefitinib plus anlotinib	48,343.33	-	2.74	-	1.60	-	36,366.18	-
Gefitinib plus placebo	42,524.74	-	2.50	-	1.44	-	-	

PAP, patient assistance program; LYs, life years; QALYs, quality-adjusted life years. “-” indicates that scenario analyses for price reductions were conditionally simulated based on the 5-year time horizon.

While first-line treatment with gefitinib plus anlotinib successfully delays the onset of targeted therapy resistance and precisely identifies the dominant patient population for stage IIIB–IV EGFR-mutated NSCLC in China, its substantial incremental cost does not commensurate with its marginal clinical gains under baseline unassisted conditions. To achieve cost-effectiveness, it is highly recommended that the National Healthcare Security Administration (NHSA) engage in centralized price negotiations with suppliers to lower the cost of anlotinib. This strategic price adjustment would ensure that the ICER of the first-line gefitinib-anlotinib combination falls below the designated willingness-to-pay (WTP) threshold of $38,043.34/QALY. Clinically, the utilization of this dual-oral regimen not only significantly enhances patient medication adherence but also effectively alleviates the financial constraints for eligible individuals, while simultaneously delaying the onset of therapeutic resistance in lung cancer management. Given that the progression-free survival utility score and the unit cost of anlotinib are established as the two key drivers that most significantly influence the ICER (Figure 2), implementing a PAP or strategically optimizing anlotinib pricing represents a highly effective and definitive approach to integrating this novel first-line regimen into the therapeutic landscape for Chinese patients with stage IIIB–IV EGFR-mutant NSCLC.

The findings of this study indicate that the economic value of the first-line gefitinib-anlotinib combination is not superior to that of gefitinib plus placebo. In the base-case analysis (Table 3), gefitinib monotherapy represents a more economically viable alternative; although it yields fewer QALYs than the combination arm, its substantial cost savings outweigh the marginal survival benefits offered by the addition of anlotinib at the current WTP threshold. Although the cost-effectiveness of the gefitinib-anlotinib regimen is not superior to that of gefitinib plus placebo in the baseline analysis, a more favorable trade-off between excessive costs and QALYs can be achieved under optimized scenarios. Compared with the monotherapy control, this novel first-line regimen offers an economically viable balance between expenditures and clinical efficacy once a PAP or national drug price negotiation is integrated.

The economic conclusions regarding the gefitinib-anlotinib combination therapy demonstrate exceptional robustness under both Scenario 1 and Scenario 2–3, confirming that the regimen achieves optimal cost-effectiveness whether supported by a PAP or when the unit price of anlotinib is successfully negotiated down to ≤ 20% of its baseline value. Notably, implementing a PAP for indicated individuals yields an ICER substantially lower than that achieved through a price reduction to 20% ($6,863.62/QALY vs. $36,366.18/QALY), demonstrating a superior cost-saving efficiency despite the modest incremental health gains over the gefitinib-placebo cohort. More importantly, when the model time horizon is extended to 10 years to fully encapsulate the health benefits accrued by long-term survivors, the ICER under the PAP scenario further plummets to $3,932.37/QALY. This demonstrates that under the “initial loading followed by sequential maintenance assistance” framework, as patient survival is prolonged, the proportion of cost-free medication cycles increases relative to the total treatment duration. This temporal dynamic effectively amortizes the cumulative long-term incremental costs. Ultimately, the ICER of the combination therapy under the PAP scenario represents merely 18.87% of that achieved through a 20% price reduction, falling well below the designated WTP threshold. These findings provide compelling scientific evidence that this novel first-line targeted therapy regimen can serve as a therapeutically advantageous and cost-effective option for Chinese patients with stage IIIB–IV EGFR-mutated NSCLC, provided that a PAP framework or strategic price-reduction mechanisms are successfully integrated.

A previous economic evaluation reported an ICER of $27,680.95/QALY for third-line or later-line anlotinib monotherapy vs. placebo in advanced NSCLC within China (28). In comparison, our findings demonstrated that relative to the gefitinib-placebo control, the first-line gefitinib-anlotinib regimen yielded an incremental cost of $6,863.62/QALY under the PAP framework, and $36,366.18/QALY when the price of anlotinib was slashed to 20% of its baseline cost. Both economic evaluations focused on anlotinib-based regimens for patients with advanced NSCLC in China. While prior clinical evidence highlighted that anlotinib significantly prolongs survival in Chinese patients with EGFR-mutated NSCLC across diverse histological subtypes—including refractory older adult populations with varied treatment histories—thereby establishing its efficacy in third-line or later-line settings, our study successfully expands its therapeutic application into first-line paradigms. Mechanistically, the unit cost of anlotinib alongside the utility scores for PFS and PD were identified as the key drivers dominating the model outcomes in both studies. Several reasons account for the ICER discrepancies between the two studies. First, the interventions and controls differed; the prior study evaluated anlotinib vs. placebo, whereas our model compared the gefitinib-anlotinib combination against gefitinib plus placebo, yielding dissimilarity in incremental expenditures. Second, anlotinib was applied at different therapeutic lines for the same indication. It was utilized as a third-line or later therapy in the previous study but as a first-line treatment in our cohort, leading to distinct clinical efficacy profiles and cost structures that inherently altered the resulting ICER values. Anlotinib produced 0.60 QALYs in the previous study (28), whereas the first-line gefitinib-anlotinib regimen generated 1.60 QALYs for Chinese patients with stage IIIB–IV EGFR-mutant NSCLC in our study. Given these discrepancies in the therapeutic lines, overall costs, and QALY outputs, the conclusions of the previous study are not directly comparable to ours (28). Additionally, our findings suggest that anlotinib provides superior QALY gains as a first-line therapy for advanced non-small cell lung cancer in China compared with its utilization in third-line or later-line settings.

Gefitinib combined with anlotinib provides significant clinical benefits for patients with stage IIIB–IV EGFR-mutated NSCLC and represents an effective regimen to delay targeted therapy resistance. To enhance the accessibility and cost-effectiveness of this treatment, reducing anlotinib's price to 20% of its original cost is recommended. Additionally, implementing a PAP could substantially promote its utilization as a first-line therapy for stage IIIB–IV EGFR-mutated NSCLC in China.

This study has several limitations. First, owing to the absence of definitive data on subsequent therapies in the FL-ALTER trial, we assumed that patients in both cohorts would receive similar treatment patterns after disease progression (9, 29, 30). Second, due to the lack of direct quality-of-life data, PFS utility values were derived from the third-line ALTER 0303 trial (28). Although health utilities for third-line patients are typically lower than those for first-line cohorts, adopting this parameter represents a “conservative estimate” strategy. Consequently, even under this framework—which potentially underestimates the health benefits of the combination arm—our core conclusions remain robust across all scenarios, further reinforcing the reliability of the model outcomes. Third, long-term survival data from the FL-ALTER trial remain subject to inherent uncertainty, requiring continuous clinical updates to validate our model projections. Additionally, biomarker-specific subgroup analyses were precluded by the lack of granular parameters, such as stratified survival outcomes and time-dependent transition probabilities. Despite these constraints, this study rigorously demonstrates the clinical value of first-line gefitinib plus anlotinib for stage IIIB–IV EGFR-mutated NSCLC in China. Given that the FL-ALTER study did not report stepwise dose reductions (from 12 mg to 10 mg and then to 8 mg), we applied a conservative boundary assumption where all patients eventually transitioned to the 8 mg dose. Upon recalculation, the ICER increased from $208,618.86/QALY to $418,046.00/QALY, consistently remaining well above the WTP threshold of $38,043.34/QALY. Additionally, dynamic ctDNA testing is currently excluded from China's National Reimbursement Drug List, and its costs were omitted from this study. However, the budget impact of this exclusion is negligible and does not alter the core conclusion that the gefitinib-anlotinib combination lacks a cost-effectiveness advantage under the current Chinese WTP threshold, regardless of the diverse therapeutic regimens utilized in real-world clinical practice. Future studies should evaluate the gefitinib-anlotinib combination against other commonly utilized real-world therapeutic strategies to provide a more comprehensive economic evaluation.

## Conclusions

At a WTP threshold of $38,043.34/QALY, gefitinib combined with anlotinib is cost-effective for Chinese patients with stage IIIB–IV EGFR-mutated NSCLC, provided that a PAP is implemented or the price of anlotinib is reduced to ≤ 20% of its baseline cost. These findings underscore the importance of reducing the price of anlotinib through PAPs or National Reimbursement Drug List negotiations in China to improve treatment accessibility and affordability. Such measures are critical for maximizing the value of novel therapeutic strategies and ensuring broader patient benefits.

## Data Availability

The datasets used and analyzed during the current study are available from the corresponding author on reasonable request.
